# Haemodynamic Vulnerability: An Integrative Physiological Framework for Understanding Hypotension in Older Adults

**DOI:** 10.3390/jcm15218720

**Published:** 2026-11-08

**Authors:** Laurentiu Stoicescu, Steliana Ghibu, Lucreția Avram, Valer Donca, Claudiu Morgovan, Dana Crișan

**Affiliations:** 1 Internal Medicine Department, Faculty of Medicine, “Iuliu Haţieganu” University of Medicine and Pharmacy, 400000 Cluj-Napoca, Romania; laurentiu.stoicescu@umfcluj.ro (L.S.); crisan.dana@umfcluj.ro (D.C.); 2 Department of Pharmacology, Physiology and Pathophysiology, Faculty of Pharmacy, Iuliu Hațieganu University of Medicine and Pharmacy Cluj-Napoca, 6A Louis Pasteur Street, 400349 Cluj-Napoca, Romania; 3 Geriatrics—Gerontology, Department 5—Medical Specialties, Faculty of Medicine, Iuliu Haţieganu University of Medicine and Pharmacy, 400012 Cluj-Napoca, Romania; avram.lucretia@umfcluj.ro (L.A.); valer.donca@umfcluj.ro (V.D.); 4 Faculty of Medicine, “Lucian Blaga” University of Sibiu, 550169 Sibiu, Romania; claudiu.morgovan@ulbsibiu.ro

**Keywords:** hypotension, orthostatic hypotension, cardiovascular ageing, haemodynamic vulnerability, haemodynamic reserve, physiological reserve, older adults

## Abstract

**Background:** Hypotension in older adults is usually classified into distinct syndromes (orthostatic, postprandial, neurogenic, medication-related and illness-associated) that differ in mechanism, yet frequently coexist and are poorly explained by resting blood pressure. **Content:** We review how structural and functional cardiovascular ageing progressively erodes integrated haemodynamic reserve across six domains: cardiac, arterial, venous (preload), autonomic, renal/neurohumoral and regional autoregulatory. On this basis, we propose haemodynamic vulnerability as a physiological framework: a latent state of reduced integrated haemodynamic reserve that could, in future studies, be identified by abnormal responses to standardized challenges falling short of the diagnostic criteria for hypotension. We distinguish it from normal cardiovascular ageing and from related concepts (physiological reserve, frailty, resilience and haemodynamic frailty), propose an operational definition with testable predictions, outline candidate indices of reserve and resilience, and consider the potential contribution of artificial intelligence. **Clinical implications:** The framework shifts attention from resting blood pressure to haemodynamic competence, the ability to maintain blood pressure and organ perfusion under stress, and helps explain the overlap between hypotensive phenotypes. **Conclusions:** Haemodynamic vulnerability links cardiovascular ageing to the different hypotensive phenotypes. Prospective validation is required before it can support risk stratification and clinical decision-making.

## 1. Introduction. Why Does Hypotension in Older Adults Matter?

Hypotension in older adults is commonly regarded as an isolated blood pressure abnormality, a transient symptom, or an adverse effect of cardiovascular therapy. This view is incomplete. In later life, a low blood pressure reading or an episodic fall in pressure often reflects impaired cardiovascular adaptation: one or more compensatory mechanisms fail to preserve adequate organ perfusion under physiological, pathological, or therapeutic stress. Standing, meal ingestion, dehydration, heat exposure, acute illness, infection, and medication changes are therefore more than precipitants of hypotension; they act as physiological challenges that unmask a diminished haemodynamic reserve.

The issue has become more pressing as populations age and cardiovascular care grows more complex. Older adults frequently present with hypertension alongside frailty, multimorbidity, polypharmacy, chronic kidney disease, diabetes mellitus, heart failure, neurodegenerative disorders, and other chronic conditions. These conditions increase the need for cardiovascular protection and, at the same time, reduce tolerance to haemodynamic stress.

Orthostatic hypotension remains the best-characterized hypotensive phenotype. It is conventionally defined as a sustained reduction in systolic blood pressure of at least 20 mmHg or in diastolic blood pressure of at least 10 mmHg within three minutes of standing or head-up tilt [1,2]. Yet it is only one part of a broader spectrum of haemodynamic dysregulation that also includes initial and delayed orthostatic hypotension, postprandial hypotension, medication-related hypotension, nocturnal hypotensive patterns, and hypotension associated with acute illness. These syndromes often coexist in the same individual and may be different clinical expressions of a shared impairment in blood pressure regulation and haemodynamic reserve (Table 1). Throughout this review, haemodynamic reserve is considered across six complementary domains (cardiac, arterial, venous/preload, autonomic, renal/neurohumoral and regional autoregulatory).

The epidemiological burden is substantial. Orthostatic hypotension is common among community-dwelling older adults and is more prevalent still in institutionalized populations and in patients with autonomic failure, Parkinsonian disorders, diabetes mellitus, cardiovascular disease, and frailty [2,3,4].

Postprandial hypotension is also common but considerably under-recognized: a recent systematic review and meta-analysis estimated its pooled prevalence among older adults at approximately 40%, ranging from about 33% in community settings to about 49% in hospital geriatric departments [5].

The clinical consequences of hypotension extend well beyond dizziness and syncope. Orthostatic and other hypotensive phenotypes have been associated with falls, cognitive impairment, functional decline, cardiovascular events, renal dysfunction, reduced quality of life, and mortality [6,7,8,9,10]. Besides contributing directly to these outcomes, hypotension may serve as a clinical marker of impaired autonomic regulation, vascular disease, cardiac dysfunction, frailty, or a multisystem loss of physiological reserve. In many patients, therefore, it reflects both impaired organ perfusion and reduced cardiovascular adaptive capacity.

**Table 1:** Hypotensive phenotypes in older adults. ABPM, ambulatory blood pressure monitoring; BP, blood pressure; DBP, diastolic blood pressure; HR, heart rate; OH, orthostatic hypotension; PDE-5, phosphodiesterase type 5; SBP, systolic blood pressure [1,2,11,12,13,14,15].

Phenotype	Operational Definition	Dominant Mechanism(s)	Typical Stressor/Trigger	Reserve Domains Mainly Challenged
Initial orthostatic hypotension	Transient fall in SBP > 40 mmHg and/or DBP > 20 mmHg within the first 15 s of active standing; requires beat-to-beat BP measurement	Temporary mismatch between cardiac output and systemic vascular resistance at the onset of active standing (vasodilatation in contracting leg muscles), with delayed baroreflex-mediated vasoconstriction	Rapid active standing (typically not reproduced by passive tilt)	Autonomic (speed of baroreflex response); arterial; cardiac
Classic orthostatic hypotension	Sustained fall in SBP ≥ 20 mmHg and/or DBP ≥ 10 mmHg within 3 min of standing or head-up tilt (≥60°); ≥30/15 mmHg suggested when supine hypertension is present	Insufficient compensatory vasoconstriction and/or cardiac output in response to reduced venous return; often multifactorial (autonomic, volume, drug, cardiac)	Standing, prolonged upright posture, morning hours, after meals or heat exposure	Autonomic; venous (preload); arterial; cardiac
Delayed orthostatic hypotension	Sustained fall meeting the BP criteria for OH occurring only after 3 min of standing or tilt	Gradual failure of vasoconstrictor compensation and progressive reduction in venous return; may represent mild or early autonomic failure	Prolonged standing (queues, standing activities)	Venous (preload); autonomic; renal/neurohumoral
Neurogenic orthostatic hypotension	OH with inadequate heart-rate increase; ΔHR/ΔSBP ratio < 0.5 bpm/mmHg at 3 min suggests a neurogenic cause	Impaired release of noradrenaline from sympathetic efferents (Parkinson disease, multiple system atrophy, pure autonomic failure, dementia with Lewy bodies, diabetic or amyloid neuropathy); frequently coexists with supine hypertension and nocturnal natriuresis	Standing, meals, heat, physical exertion, early morning; worsened by dopaminergic and vasoactive drugs	Autonomic (dominant); renal/neurohumoral; venous
Non-neurogenic orthostatic hypotension	OH with preserved compensatory heart-rate increase (ΔHR/ΔSBP ≥ 0.5 bpm/mmHg)	Volume depletion, vasoactive medication, physical deconditioning, impaired cardiac filling or output, venous pooling	Dehydration, diuretic intensification, acute illness, prolonged bed rest, new or up-titrated medication	Venous (preload); renal/neurohumoral; cardiac
Postprandial hypotension	Fall in SBP ≥ 20 mmHg within 2 h of a meal, or fall to SBP < 90 mmHg when pre-meal SBP is ≥100 mmHg	Splanchnic blood pooling not matched by adequate increases in cardiac output and peripheral vasoconstriction; modulated by gastric emptying rate, glucose absorption, and vasoactive gut peptides	Large, warm, or carbohydrate-rich meals; alcohol; antihypertensive dosing before meals	Venous (preload); autonomic; arterial; cardiac
Medication-related hypotension	Hypotension (orthostatic, postprandial, nocturnal, or at rest) temporally related to drug initiation, dose increase, or combination	Vasodilatation, volume depletion, sympatholysis, negative chronotropy; cumulative effects of drug combinations (e.g., diuretics, α-blockers, nitrates, centrally acting agents, antidepressants, antipsychotics, dopaminergic drugs, PDE-5 inhibitors)	Initiation or up-titration, polypharmacy, inappropriate timing, intercurrent dehydration or illness	Depends on drug class: arterial, venous, autonomic, cardiac, renal/neurohumoral
Nocturnal hypotension/excessive nocturnal dipping	Detected by ABPM; e.g., nocturnal SBP fall > 20% of daytime mean (extreme dipping); no universally accepted threshold for nocturnal hypotension	Sleep-related sympathetic withdrawal combined with persistent antihypertensive drug effect at the nocturnal blood pressure nadir and reduced nocturnal baroreflex compensation	Bedtime antihypertensive dosing, nocturia with upright posture at night	Arterial; autonomic; regional autoregulatory (cerebral, ocular, coronary perfusion)
Reflex (neurally mediated) hypotension/syncope, including carotid sinus syndrome	Transient hypotension and/or bradycardia due to inappropriate reflex vasodilatation and/or vagal activation; carotid sinus syndrome: symptoms reproduced by carotid sinus massage with asystole > 3 s and/or SBP fall > 50 mmHg	Inappropriate reflex withdrawal of sympathetic vasoconstrictor tone (vasodepressor) and/or vagally mediated bradycardia (cardioinhibitory); carotid sinus hypersensitivity increases with age and is more frequent in fallers	Prolonged standing, heat, pain, emotional stress, micturition, cough, defecation (situational); head turning, tight collars, neck pressure (carotid sinus)	Autonomic (reflex dysregulation rather than insufficiency); cardiac (in cardioinhibitory forms)
Illness-associated hypotension	Hypotension during acute illness, not explained by a chronic phenotype alone	Hypovolaemia, inflammatory vasodilatation, impaired renal and neurohumoral volume restoration, reduced oral intake, myocardial depression	Infection/sepsis, fever, gastrointestinal losses, bleeding, heat waves, surgery	Renal/neurohumoral; venous (preload); arterial; cardiac

The distinction matters because identical blood pressure values can have very different clinical consequences. The physiological significance of blood pressure depends less on its absolute value than on the relationship between arterial pressure, tissue perfusion, and the individual’s remaining haemodynamic reserve. A systolic blood pressure of approximately 100 mmHg may cause no symptoms in one older adult, yet be associated with cerebral symptoms, falls, myocardial ischaemia, or deterioration of kidney function in another. The magnitude of the pressure fall is only one determinant of clinical relevance; its duration, recovery, recurrence, and precipitating circumstances also matter, as do the compensatory responses it elicits and the vulnerability of the organs exposed to reduced perfusion.

Traditional descriptions of cardiovascular ageing have focused on structural and functional alterations in the heart, arteries, autonomic nervous system, and kidneys. When these changes are considered together, however, their most important physiological consequence is the gradual erosion of haemodynamic reserve—the capacity to preserve adequate blood pressure and organ perfusion during everyday physiological challenges. Attention thus moves from individual age-related abnormalities towards the cumulative loss of cardiovascular adaptive capacity.

We therefore introduce haemodynamic vulnerability as a physiological framework. We define it as a latent state of reduced integrated haemodynamic reserve, in which ordinary physiological, pathological, or therapeutic stressors are likely to exceed the remaining capacity to maintain blood pressure and organ perfusion. For future research, we propose that it could be identified operationally by abnormal responses to standardized challenges that do not meet the diagnostic criteria for a hypotensive phenotype (Section 2.4.4). The concept is deliberately presented as a framework rather than as a new diagnostic entity. It draws on established mechanisms of cardiovascular ageing, autonomic dysfunction, vascular dysfunction, impaired cardiac reserve, altered volume regulation, sarcopenia, frailty, multimorbidity, and medication exposure. We do not suggest that all hypotensive syndromes share a single mechanism. The aim is rather to explain why clinically distinct hypotensive phenotypes frequently coexist, and why apparently similar reductions in blood pressure may have markedly different consequences. Haemodynamic vulnerability is therefore intended to complement, not replace, existing phenotype-based classifications. At this stage, the framework is intended primarily to improve physiological understanding and to inform clinical reasoning; its use for risk stratification and clinical decision-making will require prospective validation.

The coexistence of hypertension and hypotension is a clear example. Many older adults have isolated systolic hypertension, arterial stiffness, impaired baroreflex function, and increased blood pressure variability, yet remain susceptible to cerebral, coronary, or renal hypoperfusion on standing, after meals, during acute illness, or under other haemodynamic challenges. Clinical management must therefore balance long-term cardiovascular protection against the need to preserve sufficient haemodynamic reserve for everyday physiological demands [2,16,17,18].

**Figure 1:**
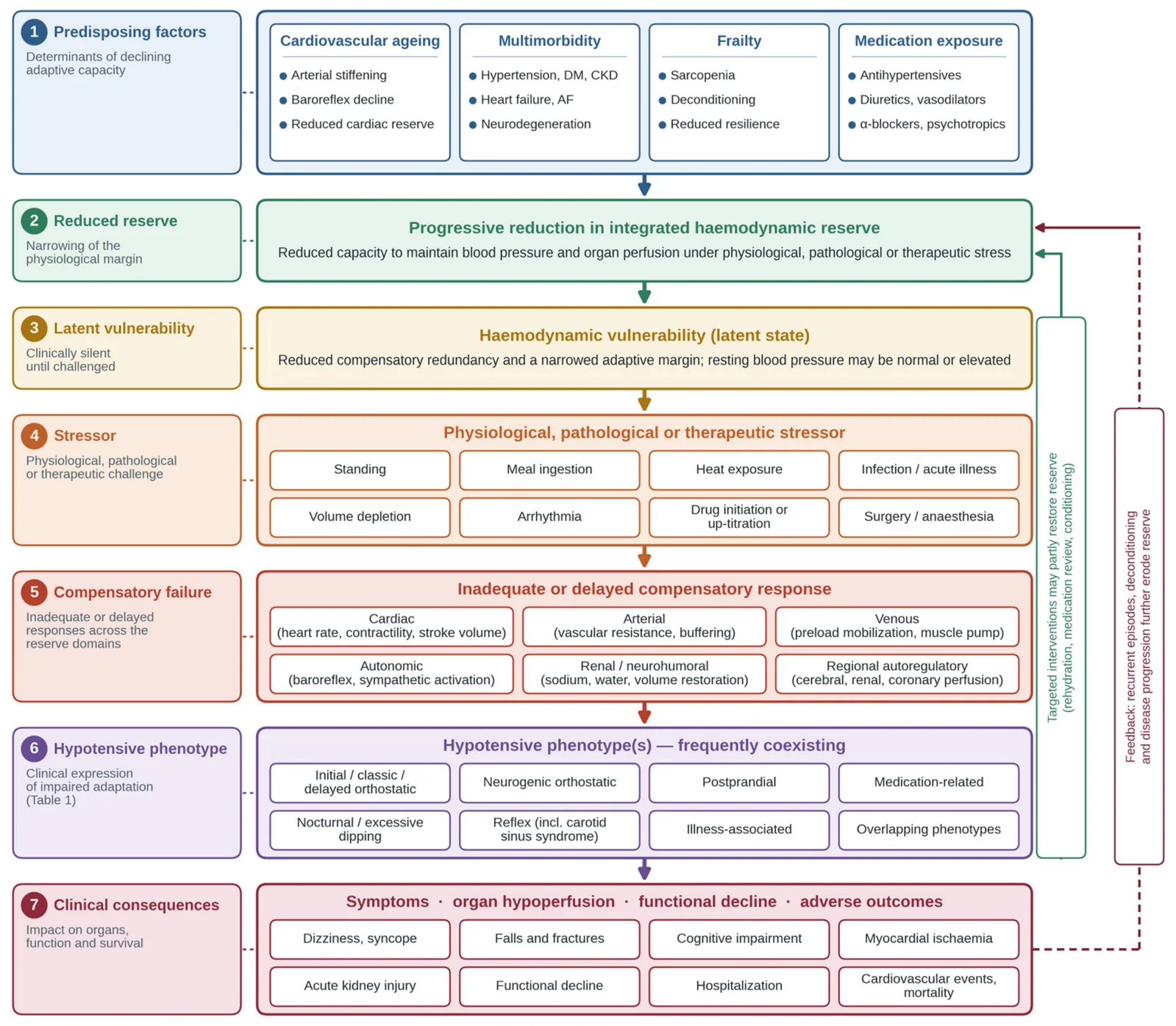
Conceptual framework of haemodynamic vulnerability in older adults. Cardiovascular ageing, multimorbidity, frailty and medication exposure progressively reduce integrated haemodynamic reserve, leading to a clinically silent state of haemodynamic vulnerability. When a physiological, pathological or therapeutic stressor exceeds the remaining adaptive capacity, compensatory responses across the reserve domains become inadequate or delayed. This results in one or more, frequently coexisting, hypotensive phenotypes and their clinical consequences. Recurrent episodes, deconditioning and disease progression further erode reserve, whereas targeted interventions may partly restore it. AF, atrial fibrillation; CKD, chronic kidney disease; DM, diabetes mellitus. The dashed red arrow indicates the feedback loop through which recurrent episodes, deconditioning and disease progression further erode reserve; the solid green arrow indicates the potential partial restoration of reserve by targeted interventions.

No single mechanism fully accounts for hypotension in older adults; it is their cumulative interaction that progressively limits the ability of the circulation to compensate for haemodynamic stress. On this view, orthostatic, postprandial, neurogenic, medication-related, and illness-associated hypotension are distinct but overlapping manifestations of impaired cardiovascular adaptation (Table 1). Their immediate mechanisms and triggers differ, but they may share a common substrate of reduced haemodynamic reserve. As shown in Figure 1, cardiovascular ageing, together with disease, frailty, and medication exposure, progressively narrows this reserve.

This review examines cardiovascular ageing as the biological substrate of the progressive loss of haemodynamic reserve. We first summarize the structural and functional changes that accompany cardiovascular ageing and then consider how autonomic dysfunction, altered volume regulation, frailty, multimorbidity, and medication exposure interact to impair circulatory adaptation to haemodynamic stress. Subsequently, we develop haemodynamic vulnerability as a framework linking these cumulative alterations to hypotension in older adults and then we discuss its implications for diagnosis, treatment, and future research, and consider how artificial intelligence might help to quantify it. This narrative review was based on a structured search of PubMed/MEDLINE, Scopus and Web of Science from inception to June–July 2026. Search terms combined “hypotension”, “orthostatic hypotension”, “postprandial hypotension” and “neurogenic orthostatic hypotension” with “ageing”, “older adults”, “cardiovascular reserve”, “baroreflex”, “arterial stiffness”, “venous return”, “cerebral autoregulation”, “frailty”, “machine learning” and “artificial intelligence”. Additional sources were identified by screening the reference lists of key articles and current guidelines and consensus statements. We prioritized guidelines, systematic reviews and meta-analyses, large cohort studies and mechanistic physiological studies in humans. Experimental and animal studies were included when they clarified underlying mechanisms. Only articles published in English were considered. Given the conceptual aim of the review, no formal quality assessment or quantitative synthesis was performed, and evidence was selected for its relevance to the proposed framework.

## 2. Cardiovascular Ageing: Progressive Erosion of Integrated Haemodynamic Reserve

### 2.1. Cardiovascular Ageing and Blood Pressure Homeostasis

Arterial blood pressure is a dynamic physiological variable rather than a static haemodynamic state. Its short-term stability relies on the coordinated interaction of several regulatory systems, including cardiac output, arterial resistance and compliance, venous capacitance, autonomic reflexes, circulating volume, renal sodium and water handling, and regional autoregulatory mechanisms. These systems maintain blood pressure and organ perfusion during everyday challenges such as postural change, meal ingestion, physical activity, and changes in environmental temperature, and must also respond to the additional stresses imposed by dehydration, blood loss, acute illness, and pharmacological interventions. Blood pressure homeostasis therefore depends not only on resting arterial pressure but on the ability of these mechanisms to generate rapid, appropriately scaled, and reversible compensatory responses to haemodynamic stress [2,19,20].

Ageing affects virtually every component of this regulatory network. Large elastic arteries stiffen, endothelial function deteriorates, left ventricular relaxation slows, ventricular and arterial systolic stiffness increases, chronotropic responsiveness declines, cardiovagal baroreflex sensitivity is attenuated, and renal regulation of sodium and water becomes less adaptable. Sarcopenia, reduced physical activity, and impaired skeletal muscle pump function further compromise venous return [20,21,22,23,24,25]. These alterations develop heterogeneously and do not inevitably culminate in cardiovascular failure. Their cumulative effect is rather a gradual loss of flexibility, coordination, and efficiency in haemodynamic regulation, which narrows the physiological range over which blood pressure and organ perfusion can be maintained under stress.

Through aortic stiffening, altered wave reflection, and widening of pulse pressure, cardiovascular ageing more commonly promotes systolic hypertension than hypotension [22,26]. Yet an individual who is hypertensive while seated or supine may still fail to maintain arterial pressure on standing, after a meal, during acute illness, or under other physiological challenges. Hypertension and hypotension are thus better viewed as complementary manifestations of impaired blood pressure regulation in the ageing cardiovascular system than as opposing physiological conditions [2,27].

Arterial ageing is a major determinant of this decline in adaptive capacity. The concepts of Early Vascular Aging (EVA) and haemodynamic ageing, originally developed to explain hypertension and cardiovascular risk, are also relevant to dynamic blood pressure regulation [26].

Cardiac and renal ageing further restrict haemodynamic adaptability. Diastolic filling, chronotropic responsiveness, and the renal capacity to defend circulating volume all decline with age, so that a modest disturbance in fluid balance can have disproportionate haemodynamic consequences [21,22,25,28].

Organ perfusion adds a further layer of complexity. Cerebral autoregulation, which normally buffers fluctuations in systemic pressure, may be altered by ageing, longstanding hypertension, small-vessel disease, and neurodegeneration, with considerable variability between individuals [29,30,31].

In this sense, cardiovascular ageing is the biological substrate of hypotension rather than its direct cause. Structural remodelling progressively constrains the operating range of the cardiovascular system, whereas functional ageing reduces the speed, precision, and effectiveness of compensatory responses. Superimposed disease, frailty, physical deconditioning, and medication exposure erode adaptive capacity further and so set the threshold at which a given stressor becomes haemodynamically significant.

### 2.2. Structural Cardiovascular Ageing

Structural cardiovascular ageing involves more than anatomical remodelling. It progressively alters the mechanical properties of the cardiovascular system and diminishes its ability to maintain haemodynamic stability under physiological stress. These changes form the anatomical substrate of declining haemodynamic reserve and affect every major component of the circulation: the arterial tree, microcirculation, myocardium, atria, cardiac valves, and venous system. They result from the cumulative effects of extracellular matrix remodelling, cellular senescence, chronic low-grade inflammation, oxidative stress, calcification, and lifelong haemodynamic loading [32,33,34,35,36].

#### 2.2.1. Arterial Ageing: From Elastic Conduit to Stiffened Pressure Reservoir

Large elastic arteries are key determinants of haemodynamic reserve because they buffer pulsatile ventricular ejection and sustain continuous organ perfusion throughout the cardiac cycle. This buffering function deteriorates gradually with ageing, reducing the capacity of the arterial tree to buffer pulsatile flow and stabilize haemodynamics [26,35,36].

Arterial ageing is characterized by fragmentation of elastic fibres, increased collagen deposition and cross-linking, medial calcification, phenotypic changes in vascular smooth muscle cells, extracellular matrix remodelling, oxidative stress, chronic low-grade inflammation, and accumulation of advanced glycation end-products [33,34,35,36]. Together, these processes transform the aorta from a compliant elastic conduit into a stiff pressure reservoir, reducing arterial compliance and increasing the haemodynamic cost of maintaining circulatory stability under stress.

The consequences extend well beyond arterial stiffness itself. Reduced arterial compliance accelerates pulse-wave velocity, advances wave reflection, augments late systolic ventricular afterload, lowers diastolic pressure, and widens pulse pressure. Loss of elastic recoil also compromises coronary perfusion pressure and weakens the ability of the arterial tree to buffer beat-to-beat fluctuations in pressure and flow. The net result is a progressively narrower haemodynamic operating range within which blood pressure and organ perfusion can be maintained.

The rate of arterial ageing varies considerably between individuals, because vascular remodelling is strongly influenced by hypertension, diabetes mellitus, chronic kidney disease, smoking, and other cardiovascular risk factors. Biological vascular age therefore corresponds only partially to chronological age, and individuals of similar age may differ substantially in arterial structure, vascular function, and available haemodynamic reserve [37].

The result is an apparently paradoxical haemodynamic phenotype. At rest, arterial ageing manifests predominantly as systolic hypertension rather than hypotension, yet the same stiffened arterial system is less able to accommodate rapid haemodynamic perturbations. In a population-based cohort, aortic stiffness and central pressures were associated with orthostatic blood pressure responses [38], and a meta-analysis of 11 studies (10,611 participants) found that increased arterial stiffness was associated with 40% higher odds of orthostatic hypotension (pooled OR 1.40; 95% CI 1.28–1.54), more strongly for central than for peripheral stiffness [39]. Arterial stiffness alone, however, cannot account for the development of hypotension.

Arterial ageing is also spatially heterogeneous. Central elastic arteries stiffen to a much greater extent than peripheral muscular arteries, reducing the normal central-to-peripheral stiffness gradient and facilitating transmission of excessive pulsatile energy into low-resistance microvascular beds, particularly in the brain and kidneys [35,40]. Chronic exposure to increased pulsatility may progressively damage the microcirculation and impair local autoregulatory capacity, thereby reducing organ perfusion reserve.

#### 2.2.2. Endothelial and Microvascular Ageing

The vascular endothelium, the interface between circulating blood and the vascular wall, regulates vascular tone, regional perfusion, inflammation, and vascular homeostasis. Ageing impairs these functions through endothelial cell senescence, reduced reparative capacity, glycocalyx disruption, altered intercellular signalling, oxidative stress, and chronic low-grade inflammation [33,41,42]. Reduced nitric oxide bioavailability, together with increased production of reactive oxygen species, further limits endothelium-dependent vasodilation and the rapid matching of regional blood flow to metabolic demand. The ageing endothelium is consequently less able to adjust vascular resistance as physiological conditions change, which weakens an important component of cardiovascular adaptive capacity.

Beyond their role in atherosclerosis, the endothelium and microcirculation function as a distributed regulatory network responsible for continuous adjustment of arteriolar resistance and capillary recruitment. Structural rarefaction, reduced capillary density, endothelial dysfunction, and impaired vasomotor signalling progressively constrain the range over which regional perfusion can be maintained, particularly when systemic blood pressure falls and tissue perfusion comes to depend on intact local autoregulatory mechanisms.

#### 2.2.3. Myocardial Remodelling: Preserved Performance, Reduced Reserve

The ageing myocardium undergoes substantial structural remodelling even in the absence of overt cardiovascular disease. Progressive cardiomyocyte loss is accompanied by compensatory hypertrophy of surviving cells, increasing interstitial and replacement fibrosis, extracellular matrix remodelling, and collagen cross-linking, ultimately leading to greater myocardial stiffness and reduced ventricular compliance [25,32,43]. At the organ level, these changes are reflected by a tendency towards concentric left ventricular remodelling with an increased mass-to-volume ratio rather than chamber dilatation, although their extent varies according to sex, cumulative blood pressure exposure, body composition, physical activity, and subclinical disease burden [44,45].

Resting left ventricular ejection fraction alone does not adequately describe the haemodynamic consequences of myocardial ageing. Systolic function often remains preserved, whereas reduced ventricular compliance limits diastolic filling reserve, increases dependence on adequate preload and atrial contraction, and narrows the range of loading conditions compatible with stable cardiac output. The ageing heart is better described as a less compliant pump than as a weaker one, with a diminished ability to accommodate rapid haemodynamic changes.

The distinction bears directly on hypotension. Reductions in venous return caused by standing, dehydration, diuretic therapy, or venodilation may produce disproportionate reductions in stroke volume when ventricular filling reserve is already constrained [22,25,32].

#### 2.2.4. Atrial and Valvular Ageing

Ageing is accompanied by atrial remodelling characterized by fibrosis, extracellular matrix expansion, myocyte alterations, and, in some individuals, chamber enlargement. These structural changes are associated with progressive impairment of atrial reservoir, conduit, and contractile function, although the degree of dysfunction varies according to loading conditions and coexisting cardiovascular disease [46,47,48]. The decline matters most when a stiff left ventricle depends on atrial contraction to maintain end-diastolic volume and stroke volume. Structural atrial disease, and especially atrial fibrillation, attenuates or abolishes this compensatory mechanism and so limits cardiovascular adaptability during haemodynamic stress.

Valvular ageing, particularly calcification of the aortic valve and mitral annulus, is an active biological process driven by inflammation, lipid infiltration and osteogenic signalling rather than passive degeneration [49,50].

Mild aortic sclerosis or mitral annular calcification rarely causes hypotension directly. Haemodynamically significant valve disease, by contrast, limits the ability to augment stroke volume (aortic stenosis) or to maintain ventricular filling and forward output (mitral disease), and so reduces the capacity to compensate for concurrent disturbances in vascular tone, autonomic regulation, or intravascular volume.

Age-related calcification of the cardiac fibrous skeleton may also contribute to conduction system disease, making impaired chronotropic adaptation particularly important when stroke-volume reserve is already limited.

#### 2.2.5. Venous Ageing: The Overlooked Determinant of Preload Reserve

Age-related changes in the venous circulation have received far less attention than arterial ageing, despite their central role in cardiovascular regulation. Because most circulating blood volume resides within the venous compartment, venous capacitance and compliance are major determinants of cardiac preload and circulatory stability. During physiological or pathological stress, rapid mobilization of blood towards the central circulation is essential for sustaining ventricular filling and stroke volume. The venous system thus acts as a dynamic volume reservoir and is a key component of cardiovascular adaptive capacity.

Ageing alters this function through changes in venous wall structure, extracellular matrix remodelling, increased venous stiffness, altered smooth muscle function, and structural degeneration of venous valves. These alterations may reduce the ability of the venous circulation to mobilize blood during orthostatic stress or hypovolaemia, thereby limiting preload reserve [51,52,53,54]. Reduced limb venous compliance may, conversely, limit gravitational pooling in the legs, so its net effect on orthostatic tolerance varies between individuals [53,54].

Given the preload dependence of the stiff ageing ventricle, venous dysfunction may amplify haemodynamic vulnerability even in the absence of major arterial or myocardial abnormalities. Its contribution remains incompletely characterized, but incorporating venous function into multidimensional assessment of cardiovascular reserve may improve prediction of orthostatic and treatment-related hypotension.

#### 2.2.6. Structural Ageing as an Integrated Haemodynamic Substrate

The structural components of cardiovascular ageing are best considered as interacting determinants of cardiovascular adaptation rather than in isolation. Progressive aortic stiffening increases pulsatile load and ventricular afterload, promoting ventricular remodelling and reduced compliance [55]. The resulting dependence on preload and effective atrial contraction is compounded by atrial remodelling [56,57], while endothelial and microvascular ageing limit the capacity to regulate regional perfusion [58]. At the same time, alterations in the venous circulation restrict mobilization of central blood volume during haemodynamic stress [51,54]. Together, these structural changes progressively diminish the ability of the cardiovascular system to maintain blood pressure and organ perfusion as physiological conditions change.

The result is more than an ageing heart coupled to an ageing vasculature: it is a single cardiovascular system whose compensatory capacity is progressively declining [55]. Once the circulation is challenged by orthostatic stress, dehydration, postprandial vasodilatation, acute illness, arrhythmia, or pharmacological intervention, several compensatory limitations may become evident simultaneously [5,59,60].

Structural remodelling alone, however, does not determine the clinical phenotype. Whether haemodynamic decompensation develops depends on the interaction between structural constraints and functional mechanisms, including autonomic regulation, cardiac and vascular reserve, circulating volume, comorbidity, medication exposure, and the nature and intensity of the haemodynamic stressor [59,60].

### 2.3. Functional Cardiovascular Ageing

Blood pressure homeostasis relies on a coordinated sequence of adaptive processes: haemodynamic disturbances must be detected, integrated neural and humoral responses generated, and cardiac, vascular, and venous adjustments combined to preserve ventricular filling, sustain regional organ perfusion, and restore circulatory stability. Ageing modifies every stage of this adaptive cascade. The result is not overt cardiovascular dysfunction at rest but a gradual decline in the speed, coordination, flexibility, and effectiveness of compensatory responses, so that cardiovascular adaptation becomes increasingly fragile as physiological demands rise [20,23,61,62].

#### 2.3.1. Baroreflex Ageing: Impaired Integration Rather than Complete Failure

Effective blood pressure regulation depends on the integrity of the entire reflex arc, including arterial mechanotransduction, afferent signalling, central autonomic integration, efferent autonomic output, and the responsiveness of cardiac, arterial, and venous effectors. Ageing alters several of these components simultaneously. Reduced distensibility of the carotid sinus and aortic arch attenuates afferent mechanotransduction, while age-related neural remodelling contributes to the well-established decline in cardiovagal baroreflex sensitivity [20,23,62].

Baroreflex ageing should not, however, be interpreted as uniform failure of the entire reflex system. Cardiovagal responses are consistently attenuated, whereas sympathetic and vascular components may remain relatively preserved in some individuals. Resting sympathetic activity often increases with age despite reduced dynamic reflex responsiveness [23,63,64,65].

Clinically, compensatory responses become slower, less effective, or insufficiently coordinated. A fall in arterial pressure may appropriately trigger autonomic activation, yet the resulting adjustments in heart rate, vascular tone, and cardiac output may fail to restore haemodynamic stability promptly. In this situation, the speed of blood pressure recovery becomes as important as the magnitude of the initial decline. Delayed recovery prolongs cerebral and systemic hypoperfusion and may escape detection when orthostatic blood pressure is assessed only after one or three minutes [61,66].

#### 2.3.2. Autonomic Ageing: Sympathetic Activation Does Not Guarantee Effective Compensation

Ageing is accompanied by progressive remodelling of autonomic cardiovascular regulation. Healthy older adults generally exhibit reduced parasympathetic cardiac modulation together with increased resting sympathetic neural activity, although the magnitude of these changes is influenced by sex, physical fitness, adiposity, comorbidity, and medication exposure [65,67,68]. Heightened sympathetic activity should not, however, be equated with more effective cardiovascular compensation.

The effectiveness of autonomic compensation depends on the magnitude of sympathetic activation, but also on its timing, regional distribution, and recruitment during stress, and on its translation into appropriate cardiovascular effector responses. This translation, commonly referred to as sympathetic neurovascular transduction and, at the level of arterial pressure, sympathetic blood pressure transduction, changes with ageing and differs between men and women [64,67,68,69]. Several mechanisms may contribute to the resulting dissociation, including reduced adrenergic receptor responsiveness, impaired post-receptor signalling, vascular remodelling, endothelial dysfunction, diminished β-adrenergic cardiac responsiveness, and heterogeneous sympathetic regulation across different vascular beds [42,70,71,72,73].

Autonomic ageing is therefore better characterized by reduced autonomic flexibility than by simple autonomic deficiency. Resting sympathetic activity may be elevated, yet the capacity to generate rapid, coordinated, and haemodynamically effective cardiovascular responses during stress is diminished. This helps explain an important clinical paradox: an older adult may have hypertension and increased sympathetic activity and still tolerate orthostatic or other haemodynamic stressors poorly.

Biological sex further modifies these relationships. Experimental studies show that the relative contributions of cardiac output, sympathetic activity, and vascular conductance to blood pressure regulation differ between men and women and are further influenced by ageing and menopause [64,67,70,74,75]. These age- and sex-specific differences need to be taken into account in physiologically informed models of haemodynamic vulnerability and when translating mechanistic observations into individualized clinical assessment.

#### 2.3.3. Declining Chronotropic and Cardiac Output Reserve

Maximum heart rate declines with age, and myocardial β-adrenergic responsiveness becomes progressively impaired. These changes are best recognized for limiting exercise capacity and peak cardiac output, but they are equally relevant to blood pressure homeostasis. An ageing heart with reduced chronotropic reserve is less able to compensate for reductions in stroke volume, increasing susceptibility to orthostatic and other hypotensive stressors [76,77,78,79].

The haemodynamic consequences are highly context dependent. An inadequate heart-rate response may fail to preserve cardiac output, whereas excessive tachycardia may shorten diastolic filling time and further compromise ventricular filling, particularly in the stiff ageing ventricle. Effective cardiovascular adaptation therefore depends on the coordinated interaction of chronotropic reserve, ventricular compliance, preload, atrial function, and diastolic filling, and with ageing this integrated response becomes progressively less flexible [73,76,77,78,80].

Contractile reserve also declines, even when resting left ventricular ejection fraction is preserved. Reduced β-adrenergic responsiveness, impaired intracellular calcium handling, and progressive ventricular–arterial stiffening limit the ability of the ageing heart to augment cardiac output during adrenergic stimulation or exercise [22,76,80]. Functional cardiovascular ageing manifests as progressive exhaustion of chronotropic and cardiac output reserve as haemodynamic demands increase.

#### 2.3.4. Ventricular–Arterial Interaction and Reduced Coupling Reserve

The heart and arterial circulation function as an integrated mechanical system in which ventricular performance and arterial load are continuously matched to optimize cardiovascular efficiency. Ventricular–arterial coupling describes the relationship between ventricular contractility and arterial load and determines the efficiency of energy transfer from the heart to the circulation. This relationship is often preserved at rest, but its ability to adapt to changing haemodynamic demands (the ventricular–arterial coupling reserve) declines with ageing [72,81,82,83]. Age-related arterial stiffening increases effective arterial elastance (Ea), while left ventricular end-systolic elastance (Ees) also rises. These parallel changes may preserve conventional resting indices of ventricular–arterial coupling despite substantial alterations in cardiovascular mechanics. Progressive ventricular and arterial stiffening shifts the circulation towards a less compliant operating state, limiting its capacity to augment stroke volume and accommodate changes in preload or afterload during haemodynamic stress [22,72,81,82,84].

The clinical relevance of reduced coupling reserve becomes apparent when the circulation is challenged. Physiological and therapeutic stressors, including standing, dehydration, venodilation, and vasodilator therapy, alter ventricular filling and arterial loading conditions. Maintenance of arterial pressure then requires coordinated adjustments in preload, stroke volume, heart rate, and systemic vascular resistance. Ageing restricts the flexibility of these responses and so increases haemodynamic vulnerability [82]. Blood pressure instability may often reflect impaired ventricular–vascular interaction, in which coordinated adjustments in cardiac output, ventricular filling, and vascular resistance fail to meet the demands imposed by haemodynamic stress.

#### 2.3.5. Peripheral Vascular Responsiveness and the Declining Precision of Vasomotor Adaptation

Maintenance of arterial pressure during haemodynamic stress requires rapid and precisely coordinated regulation of vascular resistance throughout the circulation. Ageing is accompanied by endothelial dysfunction, altered vascular smooth muscle signalling, and changes in adrenergic responsiveness, but the functional consequences extend beyond a simple reduction in vasoconstrictor capacity. Basal vascular resistance often increases, vasodilator reserve declines, and the relationship between sympathetic activation and vascular response becomes less tightly coupled, reducing the precision with which vascular resistance can be redistributed during haemodynamic stress [42,65,67,68,69,85].

Exercise offers a useful model. During exercise, older adults exhibit impaired vasodilator responses in active skeletal muscle, while maintenance of arterial pressure depends on effective sympathetic vasoconstriction in inactive vascular beds. Ageing alters the balance between local metabolic vasodilatation and sympathetic vasoconstriction (functional sympatholysis). More generally, regulation of regional blood flow becomes less flexible and less precisely coordinated with advancing age [86,87].

The splanchnic circulation is a clinically relevant example of this integrated regulation. Meal ingestion redistributes a substantial volume of blood to the splanchnic vascular bed, requiring coordinated increases in cardiac output and peripheral vasoconstriction to preserve arterial pressure. In healthy older adults these compensatory mechanisms are usually sufficient. When autonomic regulation, cardiac output reserve, or peripheral vascular responsiveness is impaired, however, the same physiological redistribution of blood may be more than the circulation can compensate for, and postprandial hypotension results [14,88]. Postprandial hypotension thus exemplifies a central argument of this review: the physiological challenge is not excessive, but the remaining cardiovascular reserve is insufficient to meet normal circulatory demands.

#### 2.3.6. Functional Regulation of Venous Return and Preload Reserve

Structural changes in the ageing venous circulation were discussed above; the dynamic regulation of venous return is a distinct, functional determinant of cardiovascular adaptation [54,89,90]. Venous return depends on the interaction between circulating blood volume, venous capacitance, the pressure gradient for venous return, sympathetic venoconstriction, respiratory mechanics, and the skeletal muscle pump [24,91,92]. Together, these mechanisms sustain ventricular filling and preload reserve during physiological challenges such as standing, exercise, and redistribution of intravascular blood volume [93].

During orthostatic stress, restoration of central blood volume requires coordinated sympathetic venoconstriction together with activation of the skeletal muscle pump. Ageing, physical inactivity, sarcopenia, neurological disease, and impaired mobility progressively compromise these responses, limiting preload reserve.

Although preload reserve has been studied primarily in exercise physiology and heart failure with preserved ejection fraction, the underlying concept is equally relevant to age-related hypotension [84,93,94]. Older adults who appear clinically euvolaemic may nevertheless have limited preload reserve. Relatively modest stressors, including diuretic therapy, venodilation, prolonged bed rest, fever, or reduced oral intake, may substantially reduce the capacity to maintain venous return and ventricular filling without causing severe absolute hypovolaemia. Functional regulation of venous return is, in effect, the preload component of cardiovascular reserve.

#### 2.3.7. Ageing of Volume Homeostasis: Slower Adaptation to Fluid and Sodium Stress

Beyond the rapid cardiovascular responses described above, maintenance of haemodynamic stability also depends on renal and neurohumoral regulation of sodium and water balance. Ageing is associated with reduced renal functional reserve, impaired urinary concentrating capacity, altered thirst perception, and changes in the renin–angiotensin–aldosterone system. Older adults typically exhibit lower renin and aldosterone activity together with attenuated neurohumoral responses to sodium or volume depletion, although these alterations are strongly influenced by comorbidity and medication exposure [95,96,97,98,99].

These changes do not inevitably result in chronic hypovolaemia; rather, they reduce the speed and precision with which extracellular fluid volume is restored after physiological stress. Delayed recovery of sodium balance, impaired thirst responses, and reduced capacity to compensate for fluid depletion coexist with diminished free-water excretion and altered antidiuretic hormone regulation. Depending on the clinical context, older adults are predisposed to either volume depletion or fluid overload.

The defining feature of renal and neurohumoral ageing is therefore reduced homeostatic flexibility rather than a unidirectional disturbance of volume status. This limited adaptability becomes clinically relevant during common stressors such as gastrointestinal illness, fever, reduced food or fluid intake, intensified diuretic therapy, or heat exposure [95,96].

#### 2.3.8. Cerebral Perfusion: Systemic Pressure, Autoregulation, and Symptom Expression

The clinical consequences of systemic hypotension depend largely on whether the cerebral circulation can preserve adequate cerebral perfusion. Cerebral autoregulation comprises the dynamic mechanisms that stabilize cerebral blood flow despite changes in perfusion pressure. Cerebral blood flow is now recognized to remain partially pressure-dependent rather than held on a fixed autoregulatory plateau, and autoregulatory effectiveness is influenced by arterial carbon dioxide tension, neural activity, endothelial function, arterial stiffness, and underlying cerebrovascular disease [30,31,100,101].

The independent effect of healthy ageing on dynamic cerebral autoregulation remains uncertain. While some studies suggest relative preservation of autoregulatory function, others report impaired cerebrovascular responsiveness during orthostatic stress, acute hypotension, or other physiological challenges [100,101]. These discrepancies probably reflect differences in methodology, study populations, hypertension burden, cerebral small-vessel disease, physical fitness, and other comorbidities. Healthy ageing should therefore not be assumed to produce uniform impairment of cerebral autoregulation [30,31,102].

Clinically, the interaction between systemic haemodynamic recovery and cerebral autoregulation matters more than either process considered in isolation. During active standing, an initial fall in arterial pressure may transiently reduce cerebral blood flow or cerebral oxygenation. Whether symptoms develop depends on the combined effectiveness of systemic cardiovascular compensation and local cerebrovascular adaptation. Studies in older adults have shown that impaired maintenance of cerebral oxygenation is associated with poorer physical performance and functional status, whereas delayed orthostatic blood pressure recovery has been associated with adverse clinical outcomes in several cohorts [66,102,103].

This interaction helps explain the poor correspondence between measured hypotension and clinical symptoms. Individuals with similar reductions in brachial blood pressure may experience very different degrees of cerebral hypoperfusion because of differences in autoregulatory capacity, arterial stiffness, cerebrovascular disease, and microvascular function. Conversely, the absence of dizziness should not be interpreted as evidence of preserved cerebral perfusion [100,101]. Recurrent cerebral hypoperfusion has been proposed as a contributor to long-term cognitive decline, but current evidence is insufficient to establish a causal relationship. This hypothesis should therefore be distinguished from the well-established physiological consequences of transient cerebral hypoperfusion during acute hypotensive episodes [104,105].

#### 2.3.9. Functional Ageing as Loss of Coordinated Reserve

Although the physiological mechanisms described above are often considered individually, maintenance of blood pressure and tissue perfusion depends on their precise temporal coordination. Successful adaptation to haemodynamic stress requires rapid detection of the disturbance, effective autonomic integration, coordinated cardiac and vascular responses, preservation of ventricular filling, and adequate regional autoregulation. In older adults, clinically significant haemodynamic instability usually reflects the cumulative effect of multiple modest impairments rather than severe dysfunction of a single regulatory mechanism [60,106].

What functional ageing erodes, in other words, is the coordination between systems more than the performance of each one. Impairment of one component may be offset by preservation of others. Reduced cardiovagal baroreflex sensitivity, limited chronotropic reserve, or impaired vascular responsiveness alone need not result in hypotension if the remaining compensatory mechanisms remain effective. Conversely, modest deficits distributed across several physiological domains may collectively exceed the available adaptive capacity when an additional physiological or therapeutic stressor is superimposed [106,107].

**Figure 2:**
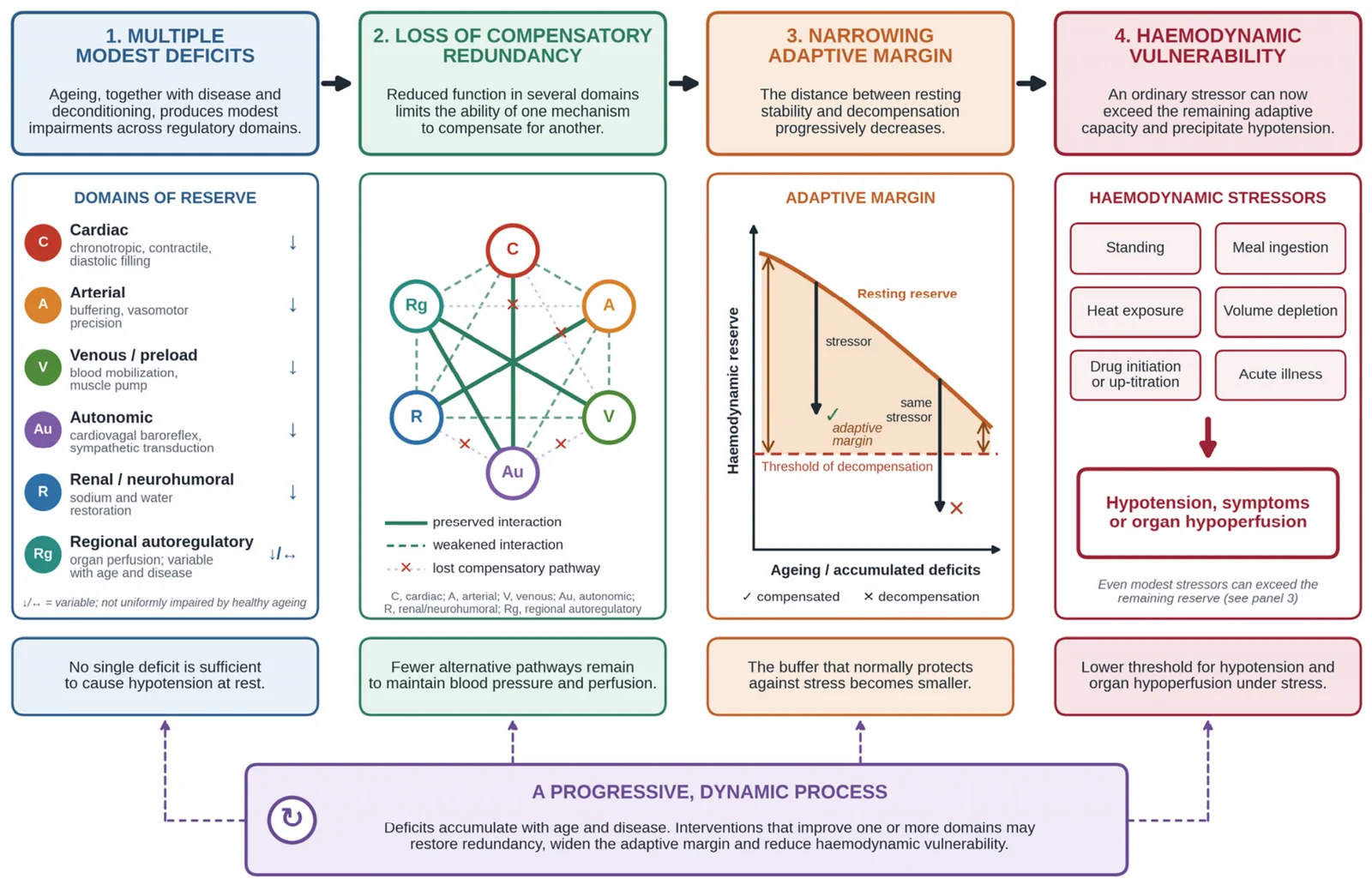
Functional cardiovascular ageing as progressive loss of coordinated compensatory reserve. (1) Ageing, disease and deconditioning produce modest impairments across the six domains of haemodynamic reserve; regional autoregulation is variably affected. (2) Concurrent deficits weaken or abolish the interactions through which one mechanism normally compensates for another. (3) Resting reserve remains above the threshold of decompensation, but the adaptive margin narrows, so that a stressor tolerated earlier in life now precipitates decompensation. (4) In this state of haemodynamic vulnerability, ordinary stressors can cause hypotension, symptoms or organ hypoperfusion. The process is dynamic and partly modifiable. A, arterial; Au, autonomic; C, cardiac; R, renal/neurohumoral; Rg, regional autoregulatory; V, venous. Dashed purple arrows indicate that the accumulation of deficits, and the effects of interventions, can act at each stage of the process.

Hypotension develops when the integrated compensatory capacity of the cardiovascular system becomes insufficient for the magnitude of the haemodynamic challenge [106]. The process is best viewed as a continuum in which multiple modest physiological deficits progressively erode compensatory redundancy, narrow the adaptive margin, and increase haemodynamic vulnerability (Figure 2).

### 2.4. From Cardiovascular Ageing to Haemodynamic Vulnerability

#### 2.4.1. Reserve, Resilience, Competence and Vulnerability

The transition from cardiovascular ageing to haemodynamic vulnerability is easier to understand if two complementary physiological concepts, reserve and resilience, are kept distinct. Physiological reserve is the functional capacity available before exposure to a stressor; resilience is the ability to resist, adapt to, and recover after that challenge [108,109,110]. Resting blood pressure reflects only the haemodynamic state at a given moment and provides limited information about either property.

Two further terms complete this vocabulary. Haemodynamic competence denotes the observed ability to maintain blood pressure and organ perfusion during a specific challenge; whereas reserve is a latent capacity, competence is its measurable expression under a defined stress. Haemodynamic vulnerability is the state in which reduced reserve makes failure of competence likely. In practice, reserve is reflected by the magnitude of the compensatory response at the peak of a challenge (for example, the increase in heart rate, stroke volume or peripheral resistance on standing), and resilience by the kinetics of recovery afterwards (for example, the time needed to restore blood pressure after standing). Although resilience is defined broadly, its resistance and adaptation components are expressed in the magnitude of the response during the challenge and therefore overlap with reserve; the resilience indices proposed in Section 2.4.4 accordingly capture its recovery component, which is the most directly measurable.

Frailty is closely related to these concepts but is a distinct clinical construct. It reflects increased vulnerability arising from cumulative decline across multiple physiological systems rather than impairment of a single cardiovascular mechanism [111]. Reserve and resilience may also dissociate: an individual with reduced physiological reserve may still demonstrate adequate resilience during a modest haemodynamic challenge, whereas another with similar resting haemodynamics may develop a disproportionate and prolonged circulatory disturbance.

The distinction is relevant to hypotension because the clinical impact of a blood pressure reduction depends on its temporal profile as well as its magnitude [9,103]. Haemodynamic vulnerability involves impairment of both reserve and resilience, which helps explain why apparently similar hypotensive episodes may have markedly different clinical consequences.

#### 2.4.2. Haemodynamic Reserve as an Integrated Physiological Capacity

The term haemodynamic reserve has traditionally been used in exercise physiology, heart failure, and invasive haemodynamic assessment to describe the capacity of the cardiovascular system to augment cardiac output, adjust filling pressures, and preserve tissue perfusion during increased metabolic or circulatory demand. This reserve declines with ageing, but the traditional concept primarily reflects cardiovascular performance during exercise and does not fully capture the integrated adaptations required to maintain blood pressure and organ perfusion during everyday physiological stress [76,80,94].

In this review, haemodynamic reserve is defined more broadly as the integrated physiological capacity of the cardiovascular, autonomic, renal, and regional circulatory systems to preserve haemodynamic stability and adequate organ perfusion during physiological, pathological, or therapeutic stress. Defined in this way, it is a composite physiological construct rather than a single measurable variable or biomarker.

**Figure 3:**
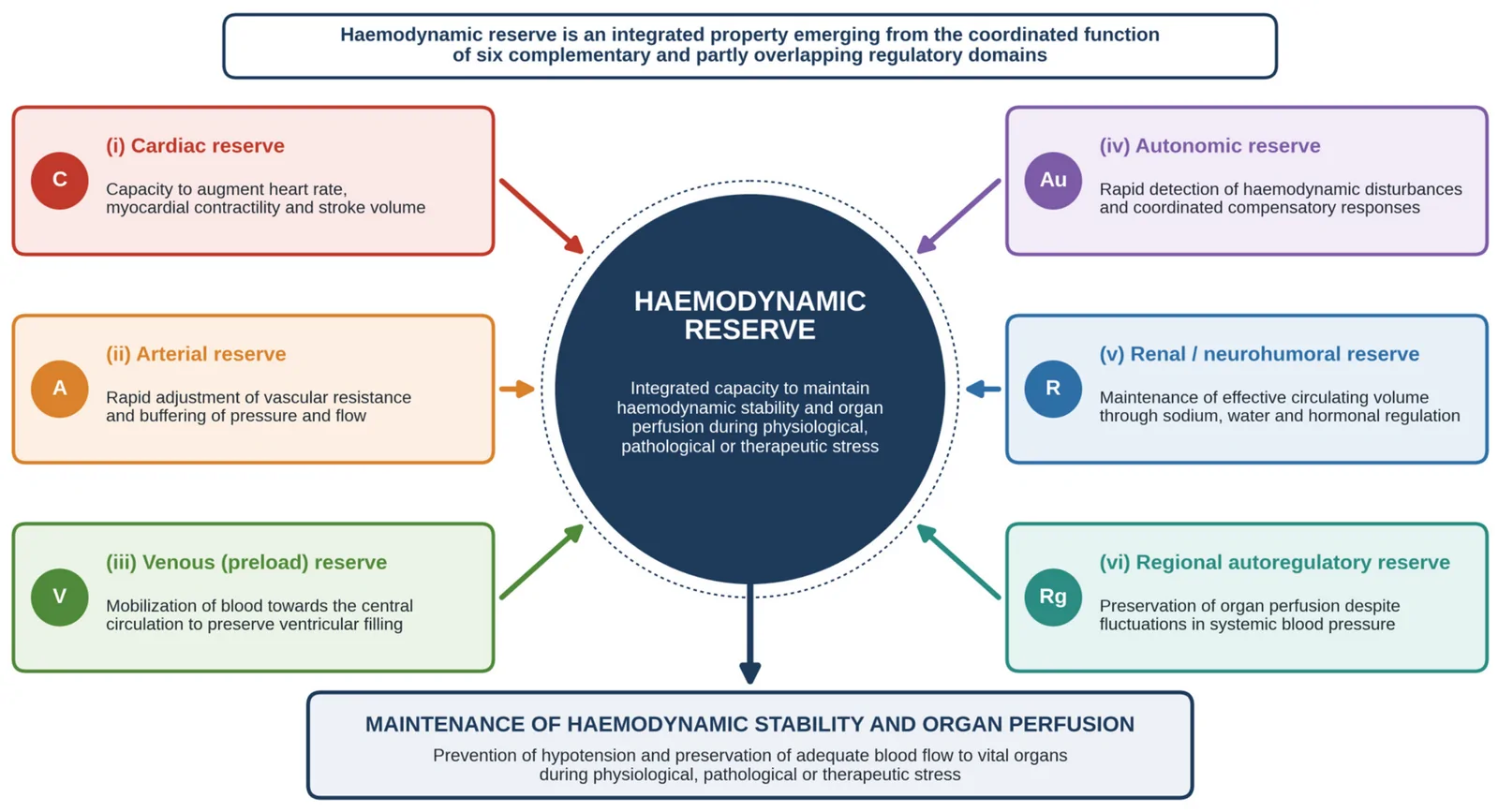
Haemodynamic reserve as an integrated physiological capacity: six complementary domains proposed in this review.

We propose that haemodynamic reserve comprises six complementary physiological domains (Figure 3):

(i) Cardiac reserve, determining the capacity to augment heart rate, myocardial contractility, and stroke volume.
(ii) Arterial reserve, enabling rapid adjustment of vascular resistance and buffering fluctuations in pressure and flow.
(iii) Venous (preload) reserve, preserving ventricular filling through mobilization of central blood volume.
(iv) Autonomic reserve, providing rapid detection of haemodynamic disturbances and coordinated cardiovascular compensation.
(v) Renal and neurohumoral reserve, maintaining effective circulating volume through integrated sodium, water, and neurohumoral regulation.
(vi) Regional autoregulatory reserve, preserving organ perfusion despite fluctuations in systemic blood pressure.

The domains most challenged in each hypotensive phenotype are summarized in Table 1. Because the domains overlap, preservation of one may partly offset impairment of another.

#### 2.4.3. From Reduced Reserve to Haemodynamic Vulnerability

Building on the related concept of haemodynamic frailty, we propose haemodynamic vulnerability as a physiological framework for the intermediate state between cardiovascular ageing and clinically overt hypotension [10]. As defined in the Introduction section, the term denotes a latent state of reduced integrated haemodynamic reserve in which ordinary stressors are likely to exceed the remaining capacity to maintain blood pressure and organ perfusion; its purpose is mechanistic rather than diagnostic.

Haemodynamic vulnerability builds on several established concepts, which are compared in Table 2. Lipsitz’s work on the dynamics of stability [106] showed that ageing reduces the capacity of regulatory systems to respond to perturbation; it provides the general principle but is not organ-specific and does not identify which cardiovascular mechanisms fail or how they could be measured. Frailty and physical resilience [108,109,110,111] describe whole-organism vulnerability and recovery, with functional outcomes such as falls, disability and death rather than haemodynamic ones. Haemodynamic frailty [10] is the closest antecedent: it links age-related cardiovascular changes to acute kidney injury during haemodynamic stress, but it focuses on a single organ outcome, mainly in acute and perioperative settings. The present proposal applies these principles specifically to blood pressure regulation in three ways: it decomposes haemodynamic reserve into six mechanistically distinct domains; it links impairment of these domains to specific hypotensive phenotypes (Table 1); and it maps each domain to available assessment tools. Its specific contribution is therefore to translate these general principles into a cardiovascular framework in which mechanisms, clinical phenotypes and measurement are explicitly linked and can be tested.

Haemodynamic vulnerability is not defined by age but by the relationship between remaining reserve and the stressors an individual is likely to encounter. Age-related decline in cardiovascular function reduces reserve, but competence is preserved as long as ordinary stressors such as standing, meals and usual medication are tolerated without failure. Three states can therefore be distinguished along a continuum: normal cardiovascular ageing, in which reserve is reduced but responses to standardized challenges remain within the normal range; haemodynamic vulnerability, in which responses to standardized challenges are abnormal but do not meet the diagnostic criteria for a hypotensive phenotype (Section 2.4.4); and overt hypotension, in which ordinary stressors, or tests that reproduce them, produce hypotension that meets established diagnostic criteria. Responses to standardized tests require careful interpretation. Hypotension that meets established diagnostic criteria during a test that reproduces an ordinary daily stressor, such as classic orthostatic hypotension on active standing or postprandial hypotension after a standard meal, indicates an established hypotensive phenotype, even if no hypotensive episodes have been recognized in daily life, because such episodes are frequently asymptomatic or unreported. Haemodynamic vulnerability, by contrast, refers to responses that are abnormal but do not meet these criteria, such as delayed blood pressure recovery, an inadequate heart-rate response or reduced baroreflex function, or to hypotension that occurs only under challenges exceeding usual daily demands, such as prolonged head-up tilt or lower-body negative pressure. The thresholds separating these states will need to be derived empirically, using age- and sex-specific reference values for each domain.

These concepts can be brought together in a single physiological continuum (Figure 1). Cardiovascular ageing progressively impairs structural and functional reserve, reduces compensatory redundancy, and narrows the adaptive margin available to sustain circulatory stability. When stress exceeds the remaining reserve, haemodynamic vulnerability becomes clinically manifest through impaired compensation, delayed recovery, or overt hypotension, potentially leading to clinically significant organ hypoperfusion [59,66,103].

The process is neither unidirectional nor necessarily irreversible. Recurrent hypotensive episodes may further diminish adaptive reserve through physical deconditioning, reduced mobility, renal dysfunction, or therapeutic de-intensification, whereas rehabilitation, optimization of medical therapy, correction of volume depletion, and improved physical conditioning may partially restore cardiovascular adaptability. Haemodynamic vulnerability is therefore a dynamic and potentially modifiable physiological state, not an inevitable consequence of ageing [10,59,113,114,115,116].

A central implication of this framework is that haemodynamic vulnerability is not synonymous with low resting blood pressure. Individuals with relatively low resting blood pressure may retain adequate compensatory reserve, whereas others with normal or even elevated office blood pressure may exhibit marked vulnerability during standing, meal ingestion, acute illness, overnight, or after treatment intensification. Resting blood pressure reflects haemodynamic status; haemodynamic vulnerability reflects a loss of haemodynamic competence [5,27,117,118].

Assessment should therefore extend beyond static blood pressure measurement to evaluate cardiovascular adaptation during physiological or clinical stress. Relevant questions include how blood pressure responds to challenge, how rapidly haemodynamic stability is restored, whether symptoms accompany the pressure change, whether organ hypoperfusion develops, and which compensatory mechanisms remain preserved or have become functionally limiting. This is the physiological rationale for combining orthostatic testing, ambulatory or home blood pressure monitoring, postprandial assessment, medication review, and evaluation of hydration status, mobility, frailty, and comorbidity in older adults at risk of hypotension [27,59,103,112,119].

#### 2.4.4. Clinical and Research Implications

At present, haemodynamic vulnerability is intended primarily as a physiological framework for understanding why older adults with similar resting blood pressure differ in their tolerance of common stressors. Even at this stage, it has practical implications for clinical reasoning: it directs attention from a single blood pressure value to the response to standing and meals, to the domains most likely to be impaired, and to the stressors an individual will face, such as treatment intensification, surgery or acute illness. Its use as a tool for risk stratification and clinical decision-making is a longer-term goal that depends on prospective validation of the operational definition proposed below.

The concept of haemodynamic vulnerability has implications for both clinical practice and research. Clinically, it moves attention away from isolated blood pressure measurements and towards the interaction between the individual, the haemodynamic stressor, and the capacity of the cardiovascular system to adapt. It encourages identification of potentially reversible contributors to reduced adaptive reserve, including dehydration, excessive diuretic therapy, prolonged inactivity, physical deconditioning, and drug combinations that impair multiple compensatory mechanisms. Management should therefore aim not only to correct hypotension when it occurs but also to preserve or restore haemodynamic reserve whenever possible [10,59,115,120].

The therapeutic objective is thus broader than raising blood pressure: it is to preserve organ perfusion and functional capacity while maintaining long-term cardiovascular protection. Treatment should target the mechanisms underlying haemodynamic vulnerability rather than blood pressure alone. Depending on the predominant mechanism, this may include optimization of medication type, dose, or timing, correction of volume depletion, physical counter-manoeuvres, treatment of postprandial or neurogenic orthostatic hypotension, and careful management of coexisting supine hypertension [27,59,114].

The framework also exposes an important research gap. Individual components of haemodynamic reserve can already be assessed, but no validated multidimensional approach yet exists for quantifying integrated haemodynamic vulnerability (Table 3).

This gap has a conceptual consequence. As currently defined, haemodynamic vulnerability is a reduced capacity to maintain blood pressure under stress, and hypotension is the failure of that capacity; the construct is therefore inferred from its own consequence and, in this form, cannot be tested or refuted. To become falsifiable, vulnerability must be measured independently of, and before, the outcome it is intended to predict. For future research, we propose as a minimum operational definition an abnormal response to at least one standardized challenge assessing a reserve domain that does not meet the diagnostic criteria for a hypotensive phenotype—for example, delayed blood pressure recovery after active standing, an inadequate chronotropic response, or abnormal baroreflex indices (Table 3). This definition is intended as a starting point rather than a validated criterion. The reproducibility of single-test results, the influence of medication, and the effects of testing conditions, such as time of day, fasting state, hydration, ambient temperature and test protocol, must first be established, and repeated or combined testing is likely to be required.

The framework would be supported by four types of finding: first, prediction of hypotensive episodes during naturally occurring stressors (drug initiation or up-titration, acute illness, surgery) by reserve measures obtained beforehand, independently of resting blood pressure, age and frailty; second, a graded relationship between the number of impaired domains and risk; third, reduced risk after targeted improvement of an impaired domain; and fourth, in experimental studies using graded orthostatic stress such as head-up tilt or lower-body negative pressure, better prediction of tolerance by combined multi-domain deficits than by any single domain. Conversely, the framework would be weakened if reserve measures added no predictive information beyond resting blood pressure and frailty, if tolerance were explained entirely by a single domain, or if domain measures were so strongly correlated that the six-domain structure could not be distinguished empirically.

Future studies should determine whether multidimensional assessment of haemodynamic reserve improves prediction of clinically meaningful outcomes (functional decline, falls, syncope, cognitive impairment, acute kidney injury, cardiovascular events, hospitalization, loss of independence, and mortality) beyond conventional blood pressure measurement.

## 3. Artificial Intelligence and Digital Phenotyping of Haemodynamic Vulnerability

Because haemodynamic vulnerability arises from interactions among several regulatory systems and depends on context, isolated measurements such as office blood pressure or a single orthostatic test capture only part of it. Machine-learning methods can integrate heterogeneous physiological and clinical data, model nonlinear relationships and analyse temporal patterns, which makes them potentially suited to characterizing it.

Early studies have shown that AI can identify orthostatic hypotension, characterize orthostatic haemodynamic responses, and detect autonomic dysfunction from combinations of demographic, haemodynamic, and autonomic variables [121,122,123,124].

Studies based on ambulatory monitoring and perioperative physiological signals show that clinically relevant information may be contained in evolving cardiovascular signals before conventional diagnostic thresholds are reached [122,125,126].

### 3.1. Current Evidence: AI in Orthostatic and Hypotension-Related Phenotyping

Clinical applications of AI in disorders of blood pressure regulation are still at an early stage. Most studies have focused on well-defined clinical entities, such as orthostatic hypotension, autonomic failure, orthostatic intolerance, or hypotensive responses to specific therapies. These investigations have addressed mainly diagnostic classification or event prediction rather than cardiovascular adaptive capacity itself, but they show that AI can extract physiologically meaningful information from complex haemodynamic data. No published study has yet evaluated haemodynamic vulnerability as the integrated physiological construct proposed here.

Early machine-learning models showed that combining demographic characteristics, resting haemodynamics, heart-rate responses, and autonomic variables identifies orthostatic hypotension better than isolated clinical parameters [124]. Subsequent studies analysed continuous blood pressure and heart-rate recordings during orthostatic testing and found that AI can identify response patterns that extend beyond conventional diagnostic categories and give insight into different mechanisms of circulatory adaptation [123].

More recent work has moved from laboratory testing to longitudinal physiological monitoring. In ambulatory blood pressure recordings, machine-learning analysis that integrated circadian profiles, variability, and hypotensive episodes recognized autonomic failure better than individual ambulatory variables [122]. Resting heart-rate variability recorded in the supine position in patients with REM sleep behaviour disorder has predicted the result of a subsequent orthostatic test, suggesting that orthostatic hypotension may be screened for without an orthostatic challenge [121]. Other models have predicted hypotensive responses after levodopa administration, an indication that AI may estimate an individual’s susceptibility to a defined physiological or therapeutic stressor, rather than simply detect an established hypotensive phenotype [127].

Unsupervised learning has taken this further by identifying physiological phenotypes without predefined diagnostic categories. Clustering of continuous cardiovascular recordings has revealed distinct orthostatic response profiles associated with frailty, sarcopenia, gait impairment, cognitive dysfunction, and falls; notably, frailty-related features clustered most in participants with a sustained orthostatic rise in blood pressure rather than a fall, indicating that the clinical significance of a blood pressure response depends on its physiological and functional context [128]. An exploratory multi-omics study in adults aged 90 years and older suggests that orthostatic hypotension involves interactions among metabolic, inflammatory, neuromuscular, neurohumoral, and neural pathways, consistent with the view that haemodynamic instability arises from multisystem biological processes rather than a single pathological mechanism [129].

These studies trace a shift in AI applications from diagnostic classification towards physiological phenotyping, longitudinal monitoring, prediction of haemodynamic responses, and multidomain characterization of cardiovascular regulation. The evidence is nonetheless limited by small or disease-specific cohorts, heterogeneous reference standards, infrequent external validation, and an emphasis on diagnostic accuracy rather than clinically meaningful outcomes. High predictive performance should not be taken as evidence that current models capture the physiological mechanisms underlying haemodynamic vulnerability.

### 3.2. Future Directions for AI-Based Assessment

The concept of haemodynamic vulnerability points to a broader objective for future AI research: to estimate the remaining adaptive capacity of the cardiovascular system, and the likelihood that compensatory mechanisms will fail under specific conditions, rather than simply to detect hypotension.

This will require models that integrate the multiple determinants of haemodynamic reserve, including cardiovascular performance, autonomic regulation, vascular adaptation, volume homeostasis, medication exposure, frailty, physical resilience, and comorbidity (Table 3). Because haemodynamic vulnerability changes over time, assessment should move beyond isolated measurements towards longitudinal monitoring with ambulatory blood pressure devices, wearable sensors, and other digital health technologies [130,131,132].

Future models should be judged by their ability to predict clinically meaningful outcomes, not hypotension alone [132]. Physiological interpretability is equally important. Beyond estimating risk, AI should identify the mechanisms most likely to limit cardiovascular compensation, such as autonomic dysfunction, impaired chronotropic reserve, reduced preload mobilization, arterial stiffening, volume depletion, or medication-related impairment. Mechanistic transparency of this kind would allow AI to support clinical reasoning and guide individualized therapeutic decisions, rather than serve solely as a predictive tool [133].

Clinical implementation will require prospective multicentre validation, standardized haemodynamic phenotypes, external validation across diverse healthcare settings, and evidence of incremental clinical value over current practice. AI should be regarded as a decision-support tool that augments, and does not replace, clinical judgement [130,131,132,133].

## 4. Reframing Hypotension in Older Adults: Implications, Limitations and Future Directions

This review proposes a reinterpretation of hypotension in older adults. Rather than an isolated blood pressure abnormality or a collection of independent clinical syndromes, many hypotensive phenotypes may be different clinical expressions of a common pathophysiological process: progressive loss of integrated cardiovascular adaptive capacity. In this model, haemodynamic vulnerability links cardiovascular ageing to the heterogeneous manifestations of hypotension encountered in clinical practice.

The model also moves attention from disease-centred thinking towards stress physiology. Common stressors become probes of adaptive capacity, not merely causes of hypotension. Its relationship to frailty, physical resilience, the dynamics of stability and haemodynamic frailty is summarized in Section 2.4.3 and Table 2.

Several limitations should be acknowledged. Haemodynamic vulnerability remains a conceptual physiological construct rather than a validated clinical entity. There are as yet no standardized diagnostic criteria, multidimensional assessment tool, or quantitative index. In its current form, the construct is also partly defined by its own consequence, and it becomes testable only when measured independently of the hypotensive outcome, as proposed in Section 2.4.4. The relative contribution of individual physiological domains is also likely to vary with age, sex, comorbidity, frailty, cardiovascular disease, autonomic dysfunction, and the nature of the precipitating stressor. This is also a narrative rather than a systematic review, so the selection of evidence may be subject to bias. The model should therefore be regarded as hypothesis-generating, intended to stimulate validation studies rather than to replace established diagnostic classifications.

## 5. Conclusions

Hypotension in older adults is better understood as a manifestation of impaired cardiovascular adaptation than simply as a disorder of blood pressure. In this review, we propose haemodynamic vulnerability as a physiological framework linking cardiovascular ageing to the heterogeneous clinical expression of hypotension. We define it as a latent state of reduced integrated haemodynamic reserve, potentially identifiable by abnormal but subthreshold responses to standardized challenges, a criterion that requires validation in future research. Hypotension is neither an inevitable consequence of ageing nor the failure of a single regulatory mechanism; it emerges when the remaining haemodynamic reserve is no longer sufficient to preserve blood pressure and organ perfusion under stress.

The framework distinguishes haemodynamic status from haemodynamic competence. Resting blood pressure reflects the haemodynamic state of the circulation, whereas haemodynamic competence describes its capacity to maintain circulatory stability under stress. This distinction helps explain why older adults with similar resting blood pressure vary so markedly in susceptibility to hypotension, symptom burden, and clinical outcomes.

Haemodynamic vulnerability remains a conceptual framework that requires prospective validation, but it gives a biological rationale for future research and clinical practice. Advances in multimodal physiological assessment, wearable technologies, and artificial intelligence may allow objective quantification of haemodynamic reserve and support more personalized prediction, prevention, and management of hypotension in older adults.

The ultimate goal is not only to measure blood pressure accurately, but to determine whether the ageing cardiovascular system retains enough adaptive capacity to preserve organ perfusion when physiological demands increase.

## Figures and Tables

**Table 2:** Haemodynamic vulnerability and related concepts [10,106,108,109,110,111,112].

Concept	Definition and Scope	Relation to Stress	Main Outcomes and Measurement	Intended Use
Physiological reserve [112]	Capacity of organ systems to exceed resting function when demand increases; any organ system	Sets the maximum stress that can be tolerated	Organ-specific; maximal or provocative testing	Physiology; clinical testing
Dynamics of stability/loss of complexity [106]	Age-related reduction in the complexity and adaptability of physiological regulation; regulatory systems in general	Reduced capacity to adapt to perturbations	Instability, functional decline; nonlinear analysis of physiological signals	Mechanistic research
Frailty [111]	Multisystem decline in reserve and resistance to stressors; whole organism	Disproportionate response to minor stressors	Falls, disability, hospitalization, death; frailty phenotype or frailty index	Clinical risk stratification
Physical resilience [108,109,110]	Ability to resist or recover from functional decline after a stressor; whole organism	Defined by the response to, and recovery from, a stressor	Functional recovery; recovery trajectories after stressors	Research; prognosis
Haemodynamic frailty [10]	Age-related cardiovascular and renal changes that increase susceptibility to haemodynamic insults; cardiovascular–renal	Acute haemodynamic stress (e.g., hypotension, hypovolaemia)	Acute kidney injury; no standardized measurement	Renal and perioperative risk assessment
Haemodynamic vulnerability (this review)	Reduced integrated haemodynamic reserve, such that ordinary stressors may cause hypotension or hypoperfusion; cardiovascular system, six domains	Latent at rest; revealed by stress	Hypotensive phenotypes, organ hypoperfusion; domain-specific challenges, no validated index yet	Mechanistic framework; future risk stratification

**Table 3:** Assessment of haemodynamic reserve by physiological domain: available tools and candidate dynamic indices of reserve and resilience. ABPM, ambulatory blood pressure monitoring; BP, blood pressure; COMPASS-31, Composite Autonomic Symptom Score-31; E/e′, ratio of early mitral inflow velocity to early diastolic mitral annular velocity; ECG, electrocardiogram; eGFR, estimated glomerular filtration rate; HR, heart rate; HRV, heart-rate variability; IVC, inferior vena cava; LA, left atrial; LVEF, left ventricular ejection fraction; MIBG, metaiodobenzylguanidine; MoCA, Montreal Cognitive Assessment; MRI, magnetic resonance imaging; MSNA, muscle sympathetic nerve activity; SGLT2, sodium–glucose cotransporter 2; SPPB, Short Physical Performance Battery; STOPP/START, Screening Tool of Older Persons’ Prescriptions/Screening Tool to Alert to Right Treatment. Rv, index of reserve (magnitude of the compensatory response at the peak of a challenge); Rs, index of resilience (kinetics of recovery after the challenge). Candidate indices are proposed for future validation and are not established diagnostic criteria [35,54,66,77,101].

Reserve Domain	Physiological Target	Bedside/Routine Assessment	Specialized/Research Assessment	Candidate Dynamic Index of Reserve (Rv) or Resilience (Rs)	Main Limitations
Cardiac reserve	Ability to augment heart rate, contractility, and stroke volume; diastolic filling reserve	Heart-rate response to active standing; ECG; echocardiography (LVEF, diastolic indices such as E/e′, LA volume); natriuretic peptides	Exercise testing with chronotropic index; stress echocardiography; cardiopulmonary exercise testing; non-invasive beat-to-beat stroke volume (Modelflow, impedance cardiography) during standing	ΔHR and Δstroke volume on standing (Rv); chronotropic incompetence (<80% of heart-rate reserve achieved) (Rv)	Resting indices poorly reflect reserve; exercise testing often not feasible in frail patients; β-blockers confound HR responses
Arterial reserve	Arterial buffering capacity; ability to adjust vascular resistance	Brachial BP, pulse pressure; office and home BP variability	Carotid–femoral pulse-wave velocity; central BP and augmentation index; flow-mediated dilatation; beat-to-beat total peripheral resistance during orthostatic stress	Δ total peripheral resistance during active standing or tilt (Rv); pulse-wave velocity as structural constraint	Stiffness indices are static; vascular responsiveness requires beat-to-beat equipment
Venous (preload) reserve	Mobilization of central blood volume; skeletal muscle pump	Clinical volume assessment; signs of venous insufficiency; grip strength and gait speed (surrogates of sarcopenia and muscle-pump capacity); passive leg raise response	Venous occlusion plethysmography (capacitance, compliance); stroke-volume fall during standing or tilt; bioimpedance; IVC ultrasound	Stroke-volume decline during the first minutes of standing (Rv); stroke-volume response to passive leg raise (Rv)	Clinical assessment of volume status is unreliable in older adults; plethysmography is research-only
Autonomic reserve	Detection of haemodynamic disturbance and coordinated sympathetic/parasympathetic compensation	Active stand test with BP at 1 and 3 min; ΔHR/ΔSBP ratio; symptom questionnaires (e.g., COMPASS-31)	Ewing battery (deep breathing, Valsalva ratio and BP phases, handgrip); head-up tilt; HRV; spontaneous baroreflex sensitivity; supine and standing plasma noradrenaline; cardiac MIBG scintigraphy; microneurography (MSNA)	Adrenergic baroreflex sensitivity (Valsalva) (Rv); ΔHR/ΔSBP ratio (Rv); BP recovery after standing (Rs)	Specialized testing has limited availability; HRV is affected by arrhythmia, ectopy, and medication
Renal and neurohumoral reserve	Restoration of effective circulating volume through sodium, water, and hormonal regulation	Serum sodium, urea/creatinine ratio, eGFR; body-weight trends; orthostatic symptoms during intercurrent illness; medication review (diuretics, SGLT2 inhibitors)	Plasma and urine osmolality; renin, aldosterone, copeptin (supine and upright); fractional sodium excretion; timed day/night urine collection (nocturnal natriuresis); ABPM for nocturnal/supine hypertension	Haemodynamic and biochemical recovery after a fluid or sodium challenge (Rs; largely unexplored)	Few dynamic tests exist; values strongly modified by drugs and comorbidity
Regional autoregulatory reserve	Preservation of cerebral (and other organ) perfusion despite systemic BP changes	Symptoms during standing or after meals; cognitive screening (e.g., MoCA); falls history; renal function changes after BP lowering	Transcranial Doppler with dynamic cerebral autoregulation (transfer function analysis, thigh-cuff release); near-infrared spectroscopy of cerebral oxygenation during standing; MRI markers of small-vessel disease	Cerebral oxygenation recovery after standing (Rs); autoregulation index (Rv/Rs)	Lack of standardized protocols and normative values in older adults; symptoms are insensitive to hypoperfusion
Integrated/temporal assessment (cross-domain)	Global capacity to maintain BP under real-life stressors over time	Active stand with repeated measurements; home BP including orthostatic and postprandial readings; ABPM (nocturnal dipping, postprandial dips, hypotensive episodes, variability)	Continuous beat-to-beat active stand (nadir, time to nadir, recovery at 20–40 s); standardized postprandial test; wearable and cuffless devices (not yet validated for this purpose)	Composite of fall magnitude, time to nadir, recovery time, recurrence, and stressor specificity (Rv/Rs)	No validated composite index; limited outcome data; cuffless technologies require validation
Functional and clinical context	Vulnerability modifiers and consequences of impaired adaptation	Frailty (Clinical Frailty Scale, Fried phenotype, frailty index); SPPB, gait speed; falls and syncope history; structured medication review (e.g., STOPP/START); comorbidity burden	Longitudinal monitoring of activity, falls, and hospitalization; multidomain digital phenotyping	Change in frailty or function after hypotensive episodes (Rs)	Modifiers rather than direct measures of haemodynamic reserve

## Data Availability

No new data were created or analyzed in this study. Data sharing is not applicable to this article.

## Abbreviations

The following abbreviations are used in this manuscript:

ABPM	ambulatory blood pressure monitoring
AF	atrial fibrillation
AI	artificial intelligence
BP	blood pressure
CI	confidence interval
CKD	chronic kidney disease
COMPASS-31	Composite Autonomic Symptom Score-31
DBP	diastolic blood pressure
DM	diabetes mellitus
E/e′	ratio of early mitral inflow velocity to early diastolic mitral annular velocity
Ea	effective arterial elastance
ECG	electrocardiogram
Ees	left ventricular end-systolic elastance
eGFR	estimated glomerular filtration rate
EVA	early vascular ageing
HR	heart rate
HRV	heart-rate variability
IVC	inferior vena cava
LA	left atrial
LVEF	left ventricular ejection fraction
MIBG	metaiodobenzylguanidine
MoCA	Montreal Cognitive Assessment
MRI	magnetic resonance imaging
MSNA	muscle sympathetic nerve activity
OH	orthostatic hypotension
OR	odds ratio
PDE-5	phosphodiesterase type 5
REM	rapid eye movement
SBP	systolic blood pressure
SGLT2	sodium–glucose cotransporter 2
SPPB	Short Physical Performance Battery
STOPP/START	Screening Tool of Older Persons’ Prescriptions/Screening Tool to Alert to Right Treatment
